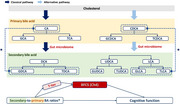# Basal forebrain cholinergic system mediates the impact of serum bile acid profiles on cognitive impairment in Alzheimer's disease

**DOI:** 10.1002/alz70855_101496

**Published:** 2025-12-23

**Authors:** Tianyi Zhang, Guoping Peng

**Affiliations:** ^1^ The first Affiliated Hospital of Zhejiang University, Hangzhou, Zhejiang, China; ^2^ The First Affiliated Hospital, Zhejiang University School of Medicine, Hangzhou, Zhejiang, China

## Abstract

**Background:**

Emerging research highlights the role of the gut microbiome in the progress of Alzheimer's disease (AD). Alterations in serum bile acid (BA) profiles, reflecting gut microbial activity, have been observed in AD patients; however, the connection to cognitive decline is still poorly understood. This research aims to deepen our understanding of the complex mechanisms through which the gut microbiome and its metabolites influence cognitive function in AD patients.

**Method:**

We analyzed data from 1,414 participants enrolled in the Alzheimer's Disease Neuroimaging Initiative (ADNI), including 389 cognitively unimpaired controls, 754 mild cognitive impairment (MCI) individuals, and 271 AD patients. We examined 15 BA metabolites and 8 BA ratios to explore their correlations with volumes of the basal forebrain cholinergic system (BFCS) and cognitive performance. We also conducted mediation analyses to assess the role of BFCS in the impact of BA profiles on cognitive function, as well as the role of AD pathology in the effect of BA profiles on BFCS.

**Result:**

Associations were observed between serum BA profiles, BFCS volumes, and cognitive performance, even after adjusting for demographic factors. The mediation analysis suggested the mediating role of the BFCS in the relationship between gut microbiota metabolism‐related secondary‐to‐primary BA ratios and cognitive function. Furthermore, the influence of secondary‐to‐primary BA ratios on BFCS was modulated by tau pathology.

**Conclusion:**

Our findings suggest that BFCS may modulate the relationship between BAs and cognitive function, with tau pathology potentially mediating the influence of BAs on BFCS. These results enhance our understanding of the intricate mechanisms through which the brain‐gut axis modulates cognitive function in AD.